# Functionalized Thallium Antimony Films as Excellent Candidates for Large-Gap Quantum Spin Hall Insulator

**DOI:** 10.1038/srep21351

**Published:** 2016-02-17

**Authors:** Run-wu Zhang, Chang-wen Zhang, Wei-xiao Ji, Sheng-shi Li, Shi-shen Yan, Ping Li, Pei-ji Wang

**Affiliations:** 1 School of Physics and Technology, University of Jinan, Jinan, Shandong, 250022, People’s Republic of China; 2 School of Physics, State Key laboratory of Crystal Materials, Shandong University, Jinan, Shandong, 250100, People’s Republic of China

## Abstract

Group III-V films are of great importance for their potential application in spintronics and quantum computing. Search for two-dimensional III-V films with a nontrivial large-gap are quite crucial for the realization of dissipationless transport edge channels using quantum spin Hall (QSH) effects. Here we use first-principles calculations to predict a class of large-gap QSH insulators in functionalized TlSb monolayers (TlSbX_2_; (X = H, F, Cl, Br, I)), with sizable bulk gaps as large as 0.22 ~ 0.40 eV. The QSH state is identified by Z_2_ topological invariant together with helical edge states induced by spin-orbit coupling (SOC). Noticeably, the inverted band gap in the nontrivial states can be effectively tuned by the electric field and strain. Additionally, these films on BN substrate also maintain a nontrivial QSH state, which harbors a Dirac cone lying within the band gap. These findings may shed new light in future design and fabrication of QSH insulators based on two-dimensional honeycomb lattices in spintronics.

One of the grand challenges in condensed matter physics and material science is to develop room-temperature electron conduction without dissipation. Two-dimensional (2D) topological insulators (TIs), namely quantum spin Hall (QSH) insulators, are new states of quantum matter with an insulating bulk and metallic edge states^1^,^2^,^3^,^4^,^5^. Their helical edge states are spin-locked due to the protection of time-reversal symmetry (TRS), namely the propagation direction of surface electrons is robustly linked to their spin orientation^6^, leading to dissipationless transport edge channels. However, the working temperature of QSH insulators in experiments like HgTe/CdTe^7^,^8^ and InAs/GaSb films^9^,^10^ are very low (below 10 K), limited by their small energy gap. The search of QSH insulators with large-gap is urgently required.

Chemical functionalization of 2D materials is an effective way to realize QSH state with desirable large-gaps. The most reported cases include hydrogenated or halogenated stanene^11^,^12^,^13^ and plumbene^14^ films. These films are QSH insulators with gaps as large as 0.2 ~ 1.34 eV, sufficient for practical applications at room temperature. Group V films, including As^15^, Bi^16^ and Sb^17^, are large-gap QSH insulators, when functionalized with hydrogen or halogens. Recently, the organic molecule ethynyl-functionalized stanene^18^,^19^ films have been reported to be good QSH insulators in the previous works. Progress also undergoes simultaneously in experiments, Bi (111) film has been successfully grown on Bi_2_Te_3_ or Bi_2_Se_3_ substrates^20^,^21^,^22^,^23^. The common feature of these materials is that they all own 2D honeycomb-like crystal structures, indicating that 2D hexagonal lattice could be an excellent cradle to breed QSH insulators with SOC. These large-gap QSH insulators are essential for realizing many exotic phenomena and for fabricating new quantum devices that can operate at room temperature.

Group III-V materials are of importance applicable to semiconductor devices in semiconductor industry. Especially, the π bonding between *p*_z_ orbitals on group-III and V atoms can generally open a bulk gap with SOC, similar to graphene^1^. Different from the inversion-symmetry (IS) in graphene, the geometry of group III-V films is not IS (inversion-asymmetry) due to IS breaking. The previous works have shown that the not IS materials host many nontrivial phenomena such as the crystalline- surface-dependent topological electronic states^24^,^25^, pyroelectricity^26^, topological *p-n* junctions^27^, as well as topological superconductivity^28^,^29^, *et al*. However, one important characteristic of III-V films is that its unsaturated *p*_z_ orbital is chemically active, due to the weak π-π interaction as caused by the bond length between III-V atoms (~3 Å). This feature, together with the out-of-plane orientation of *p*_z_ orbital, facilitates strong orbital interaction with external environments, and thus its electronic properties are easily affected by adsorbates and substrates, unfavorable for practical applications in spintroncis.

As a representative, here we provide a systematical study on structural and topological properties of 2D TlSb monolayers functionalized with hydrogen and halogens, namely TlSbX_2_ (X = H, F, Cl, Br, I). We find that the surface functionalization on TlSb, *i.e.*, saturating the *p*_z_ orbital composed of TISb with hydrogen or halogens, can stabilize the 2D TlSb, according to the calculated phonon spectrum of TlSbX_2_ films. All the systems are found to be QSH insulators, with the bulk gap in the range of 0.22 ~ 0.40 eV, tunable by external strain and electric field. A single pair of topologically protected helical edge states is established for these systems with the Dirac point locating in the bulk gap, and the odd numbers of crossings between edge states and Fermi level prove the nontrivial nature of these TlSbX_2_ films. These findings may provide a new platform to design large-gap QSH insulator based on group III-V films, which is important for device application in spintronics.

## Results and Discussion

**Figure 1:**
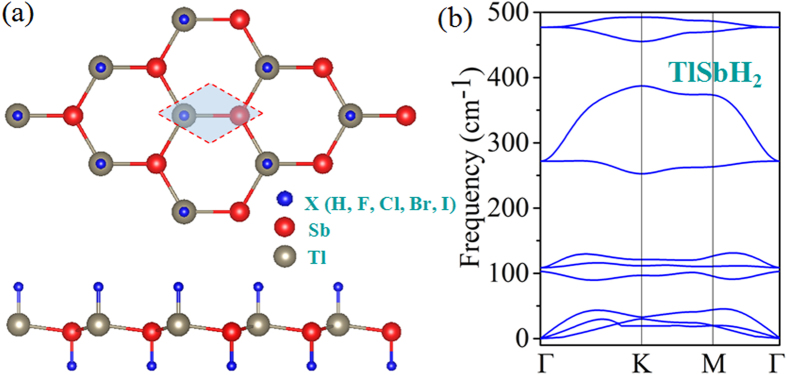
(**a**) Top and side views of the geometrical structures of TlSbX_2_ (X = H, F, Cl, Br, I). Blue, red, and gray balls denote hydrogen & halogen, Sb, and Tl atoms, respectively. Shadow area in (**a**) presents a unit cell. (**b**) Phonon band dispersion for TlSbH_2_.

The geometric structure of TlSbX_2_ (X = H, F, Cl, Br, I) are displayed in Fig. 1(a), in which the Tl or Sb atoms are saturated with X atoms on both sides of the plane in an alternating manner along the hexagonal axis, and thus breaks IS of TlSbX_2_. Table 1 lists the calculated equilibrium lattice constants, buckling heights, as well as Tl-Sb, Tl-X, and Sb-X bond lengths after structural optimization. In comparison to pristine TlSb, the TI-Sb bonds in TlSbX_2_ slightly expand, while the buckling changes differently due to the weakly hybridization between π and σ orbitals, stabilizing these structures. The stability of functionalized TlSbX_2_ is studied through the formation energy defined as


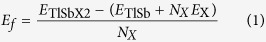


where *E*_TlSbX2_, *E*_TISb_ and *E*_X_ are the total energy of double-side functionalized TlSbX_2_, pristine TlSb, and molecule X_2_, respectively. *N*_X_ is the number of X atoms. The calculated formation energies for TlSbH_2_, TlSbF_2_, TlSbCl_2_, TlSbBr_2_, and TlSbI_2_, are −1.862, −2.997, −1.613, −1.567, and −1.420 eV, respectively, suggesting that hydrogen or halogens are chemically bonded to TlSb, indicating a higher thermodynamic stability relative to their elemental reservoirs. The dynamic stability of TlSbH_2_, as an example, is further confirmed by the phonon dispersion curves in Fig. 1(b), in which all branches have positive frequencies and no imaginary phonon modes, confirming the stability of TlSbH_2_.

**Figure 2:**
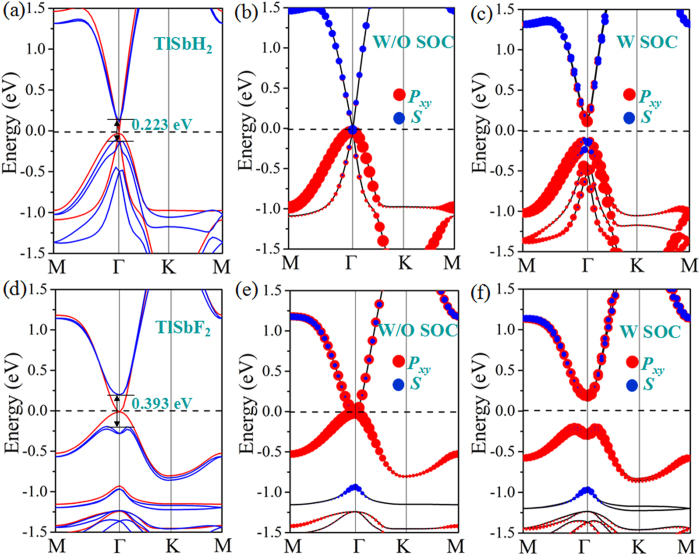
The calculated band structures for (**a**) TlSbH_2_ and (**d**) TlSbF_2_ with and without SOC. The red lines correspond to band structures without SOC, and the blue lines correspond to band structures with SOC. (**b**,**c**) Orbital-resolved band structures of TlSbH_2_, as well as (**e**,**f**) TlSbF_2_, respectively. The blue dots represent the contributions from the *s* atomic orbital, and the red dots represent contributions from the *p*_x,y_ atomic orbitals of Tl and Sb atoms.

Figure 2(a,d) display the calculated band structure for TlSbH_2_ and TlSbF_2_ as representative examples, in which the red and blue lines correspond to band structures without and with SOC. In the absence of SOC, they are both gapless semimetal with the valence band maximum (VBM) and conduction band minimum (CBM) degenerate at the Fermi level. When takes SOC into account, the band structures of TlSbH_2_ and TlSbF_2_ produce a semimetal-to-semiconductor transition, with sizeable bulk-gaps of 0.22 eV and 0.40 eV, respectively. As observed in previously reported 2D TIs like ZeTe_5_^30^, HfTe_5_^31^, and GaSe^32^, graphene-like materials^33^,^34^,^35^, the SOC-induced band-gap opening at the Fermi level is a strong indication of the existence of topologically nontrivial phases.

**Figure 3:**
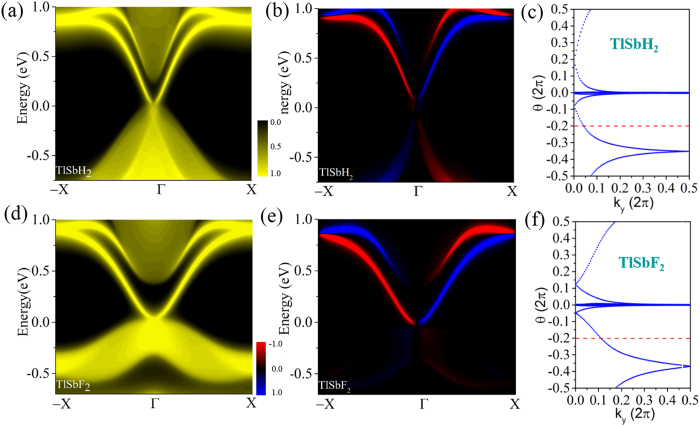
Total (left panel) and spin (right panel) edge density of states for (**a,b**) TlSbH_2_, (**d,e**) TlSbF_2_. In the spin edge plot, red/blue lines denote the spin up/down polarization. Evolutions of Wannier centers along *k*_*y*_ are presented in (**c**) TlSbH_2_ and (**f**) TlSbF_2_. The evolution lines (blue dot lines) cross the arbitrary reference line (red dash line parallel to *k*_*y*_) with an odd number of times, thus yielding Z_2_ = 1.

An important character of the QSH insulator is helical edge states which is key to spintronic applications due to the ability to conduct dissipationless currents. Thus, we calculate the topological edge states by the Wannier90 package^36^. We construct the maximally localized Wannier functions (MLWFs) and fit a tight-binding Hamiltonian with these functions. The calculated edge Green’s function^37^ of semi-infinite TlSbX_2_ (X = H, F) is shown in Fig. 3(a,d). One can see that all the edge bands connect completely the conduction and valence bands and span 2D bulk band gap, yielding a 1D gapless edge states. Besides, the counter-propagating edge states exhibit opposite spin-polarization, in accordance with the spin-momentum locking of 1D helical electrons. In addition, the Dirac point located at the band gap are calculated to have a high velocity of ~2.0 × 10^5 ^m/s, comparable to that of 5.5 × 10^5 ^m/s in HgTe/CdTe quantum well^7^,^8^. All these consistently indicate that TlSbX_2_ (X = H, F) are ideal 2D TIs. The topological states can be further confirmed by calculating topological invariant Z_2_ after the band inversion. Due to IS breaking in TlSbX_2_, the method proposed by Fu and Kane^38^ cannot be used to calculate the Z_2_ invariant. Thus, a method independent of the presence of IS is needed. As reported by Yu *et al*.^39^, we employ a recently proposed equivalent method for the Z_2_ topological invariant based on the U(2N) non-Abelian Berry connection. This approach allows the identification of the topological nature of a general band insulator without any of the gauge-fixing problems that plague the concrete, previous implementation of invariants. Here, we introduce the evolution of Wannier Center of Charges (WCCs)^39^ to calculate the Z_2_ invariant, which can be expressed as:


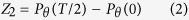


which indicates the change of time-reversal polarization (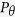) between the 0 and *T*/2. The evolution of the WCC along *ky* corresponds to the phase factor, *θ*, of the eigenvalues of the position operator,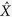, projected into the occupied subspace. Then the WFs related with lattice vector *R* can be written as:


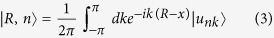


Here, a WCC 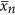 can be defined as the mean value of 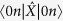, where the
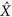
is the position operator and 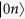 is the state corresponding to a WF in the cell with *R* = 0. Then we can obtain


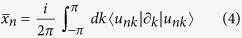


Assuming that 
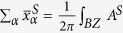
with *S* = *I* or *II*, where summation in *α* represents the occupied states and *A* is the Berry connection. So we have the format of Z_2_ invariant:


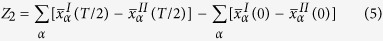


The Z_2_ invariant can be obtained by counting the even or odd number of crossings of any arbitrary horizontal reference line. In Fig. 3(c,f), we display the evolution lines of Wannier function centers (WFC) for TlSbH_2_ and TlSbF_2_, respectively. It can be seen that the WFC evolution curves cross any arbitrary reference lines odd times, thus yielding Z_2_ = 1.

**Figure 4:**
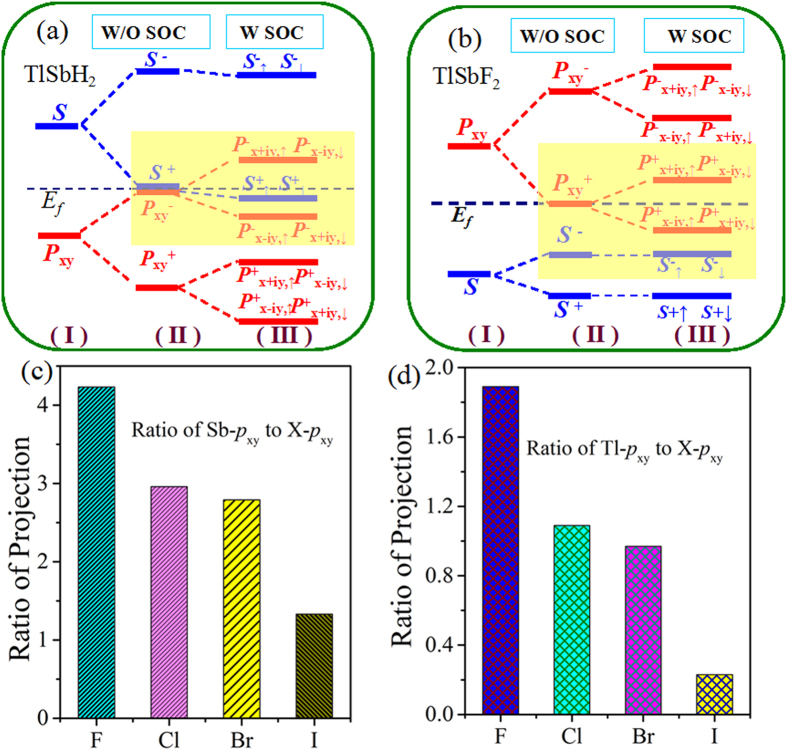
The evolution of atomic *s* and *p*_x,y_ orbitals without SOC and with SOC of (**a**) TlSbH_2_ and (**b**) TlSbF_2_. The horizontal blue dashed lines indicate the Fermi level. (**c**) The ratio of Sb-*p*_x,y_ to X-*p*_x,y_ component in the *p*_x,y_ orbital at the Fermi level. (**d**) The ratio of TI-*p*_x,y_ to X-*p*_x,y_ component in the *p*_x,y_ orbital at the Fermi level.

Now, we turn to the physics of QSH effect in TlSbX_2_. Since the decorated atoms hybridizes strongly with the dangling bonds of *p*_*z*_ orbital in TISb, it effectively removes the *p*_*z*_ bands away from the Fermi level, leaving only the *s* and *p*_x,y_ orbitals, as displayed in Fig. 2(b,c,e,f). However, through projecting the bands onto different atomic orbitals, we find that there are two scenarios for the effect of SOC on the bands around the Fermi level, in which the *s* and *p*_*x,y*_ band inversion are different from each other. For TlSbH_2_, at the Γ point, the two *p*_*x,y*_ orbitals from TI and Sb atoms are energy degenerate, while the bands away from the Γ point are well separated due to orbital splitting. The Fermi level is located between one *s* and two *p*_*x,y*_ orbitals, rendering the *s* above *p*_*x,y*_ orbitals in energy, thus forming a normal band order, similar to the cases in conventional III-V semiconductors. While for TlSbF_2_, the band structures are changed drastically, as shown in Fig. 2(d–f). In sharp contrast to TlSbH_2_, the band order at the Γ point is inverted, *i.e.*, the *s* is shifted below two *p*_*x,y*_ orbitals. These two different band orders may be attributed to the chemical bonding and orbital splitting between TI and Sb atoms. To further understand the physics of band inversion, we display in Fig. 4 the schematic of orbital inversion at the Γ point around the Fermi level in TlSbH_2_ and TlSbF_2_ films. On can see that, the chemical bonding and crystal field splitting between TI and Sb atoms make the *s* and *p*_x,y_ orbital split into the bonding and anti-bonding states, *i.e., s*^±^ and *p*^±^_x,y_, which the superscripts + and − represent the parities of corresponding states, respectively. As displayed in Fig. 4(a), the *s*^+^ orbital for hydrogenated one is significantly higher somewhere above *p*^−^_x,y_ orbital of Tl and Sb atoms under the effect of crystal field. The inclusion of SOC makes the degeneracy of *p*^−^_x,y_ orbital split into *p*^−^_x + iy,↑_& *p*^−^_x−iy,↓_ and *p*^−^_x−iy,↑_& *p*^−^_x + iy,↓_, leading *s*^+^ locate in between them. On the other hand, for TlSbF_2_ in Fig. 4(b), the larger lattice constant results in a weaker *s*-*p* hybridization, and accordingly a smaller energy separation between the bonding and anti-bonding states. Thus, the *s*^−^ orbital is downshifted while the *p*^+^_x,y_ is upshifted, *i.e.*, the *s*^−^ will be occupied, while the degenerate *P*^+^_x,y_ is half occupied, resulting in semi-metallic character (Fig. 2(d)).Though the inclusion of SOC make also the degeneracy of *p*^+^_x,y_ orbital split into *p*^+^_x + iy,↑_& *p*^+^_x−iy,↓_ and *p*_ + x−iy,↑_& *p*^+^_x + iy,↓_, but its *s-p* band order are not changed. As a result, the mechanism of QSH effect can be roughly classified into two categories: *i.e.*, type-I: SOC-induced *p-s-p* TI (TlSbH_2_), and type-II: Chemical bonding induced *p-p-s* TI (TlSbF_2_). Obviously, it is the *s* orbital insertion into *p*^+^_x + iy,↑_& *p*^+^_x−iy,↓_ and *p*^+^_x−iy,↑_& *p*^+^_x + iy,↓_ that the topological bulk-gap (0.22 eV) of TlSbH_2_ is smaller twice than that (0.40 eV) of TlSbF_2_ film.

Here, we wish to point out that fluorination in TlSb is not the only way to achieve large-gap QSH state, the same results can be obtained by decorating the surface with otherwise halogens, such as Cl, Br, and I. We thus performed calculations for TlSbX_2_ (X = Cl, Br, I) films to check their topological properties, as illustrated in Fig. S1. Table 1 summarizes their lattice constants, Sb-X and Tl-X bond lengths, and nontrivial QSH bulk-gaps at their equilibrium states. The results demonstrate that the electronic structures of all these TlSbX_2_ films are similar to TlSbF_2_, and exhibit nontrivial topological states (Fig. S2). Interestingly, as can be seen in Fig. 4(c) and Fig. S1, the global gaps in QSH state are obtained to be 0.34, 0.32, and 0.29 eV for TlSbCl_2_, TlSbBr_2_ and TlSbI_2_, respectively, which are sufficient for practical applications at room temperature. However, when comparing the band gaps with each other, we can find some fascinating phenomena that the global band gaps of these systems monotonically decrease in the contrary order of TlSbF_2_ > TlSbCl_2_ > TlSbBr_2_ > TlSbI_2_. It is known that, from F to I, the SOC becomes stronger in the order of F < Cl < Br < I, thus the SOC-induced bulk-gap should be increased correspondingly. This interesting contradiction can be attributed to the variation of band components of Tl and Sb atoms near the Fermi level, as the band splitting driven by SOC can directly determine QSH gap. As shown in Fig. 4(c)*, the ratio (R*_1_) from the Sb-*p*_x,y_ to X-*p*_x,y_ orbitals at Γ point can be established by *R*_1_ = Sb-*p*_x,y_/X-*p*_x,y_, which decreases in the order of TlSbF_2_ > TlSbCl_2_ > TlSbBr_2_ > TlSbI_2_. Similar results are obtained for the ratio *R*_2_ = TI-*p*_x,y_/X-*p*_x,y_ in Fig. 4(d). Considering that the Tl and Sb atoms exhibits stronger SOC strength than halogens, it is expected that the larger the ratio is, the larger the contribution to the states near the Fermi level, and thus the larger the SOC strength.

**Figure 5:**
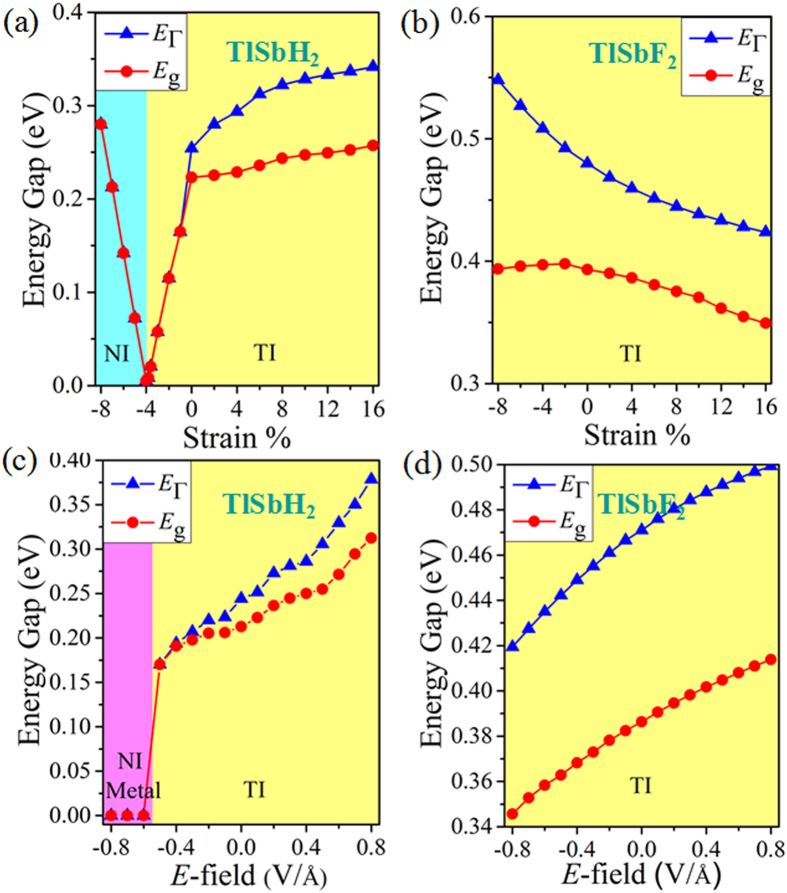
The dependence of band gap of the strain (**a**) TlSbH_2_ and (**b**) TlSbF_2_, and the electric field (**c**) TlSbH_2_ and (**d**) TlSbF_2_, respectively.

Strain engineering is a powerful approach to modulate electronic properties and topological natures in 2D materials, and thus it is interesting to study these effects in TlSbX_2_ films. We employ an external strain on these monolayers maintaining the crystal symmetry by changing its lattices as *ε* = (*a*–*a*_0_)/*a*_0_, where *a* (*a*_0_) is the strained (equilibrium) lattice constants. As shown in Fig. 5(a,b), the magnitude of nontrivial bulk-gaps of TlSbH_2_ and TlSbF_2_ can be modified significantly by strain, implying the interatomic coupling can modulate the topological natures of these systems. For TlSbH_2_, with increasing the strain, the CBM is continuously to shift downward to the Fermi level, while the VBM increases reversibly, leading the band gap to decrease significantly (Fig. 5(a)). When the critical value reaches up to −3.8%, a semi-metallic state with zero density of states at the Fermi level occurs. If increases the strain beyond −3.8%, a trivial topological phase appears. While for TlSbX_2_ (see also Fig. 5(b) and Fig. S3), both the direct and indirect band gaps decreases steadily with respect to tensile strain. Especially, these QSH states are robust with the strain in the range of −8 ~ 16%. Such robust topology against lattice deformation makes TlSbX_2_ easier for experimental realization and characterization on different substrate.

On the other hand, from the perspective of potential device applications, the ability to control topological electronic properties via the vertical electric field (*E*-field) is highly desirable. Thus, we study the change of band gaps of TlSbH_2_ and TlSbF_2_ under different vertical *E*-field, as shown in Fig. 5(c,d). One can see that the band gaps increase monotonically with increasing *E*-field strength for both cases. For TlSbF_2_ (Fig. 5(d)), under −0.8 V Å^−1^ ≤ *E*-field ≤0.8 V Å^−1^, the trend of band gaps increase monotonically from 0.34 eV to 0.41 eV, with *E*_Γ_ larger than *E*_g_ significantly. While for TlSbH_2_, when *E*-field ≤−5.5 V Å^−1^, it is a normal metal. But for large *E*-field (>−0.4 V Å^−1^), it becomes a QSH insulator, along with *E*_Γ_ being almost equal to *E*_g_. Noticeably, if *E*-field is in the range of ±8%, the nontrivial bulk-gaps of other TlSbX_2_ (X = Cl, Br, I) are still very large (~0.2−0.5 eV) (Fig. S3), allowing for viable applications at room temperature. The predicted QSH insulators tuned by vertical *E*-field may provide a platform for realizing topological field-effect transistor (TFET).

**Figure 6:**
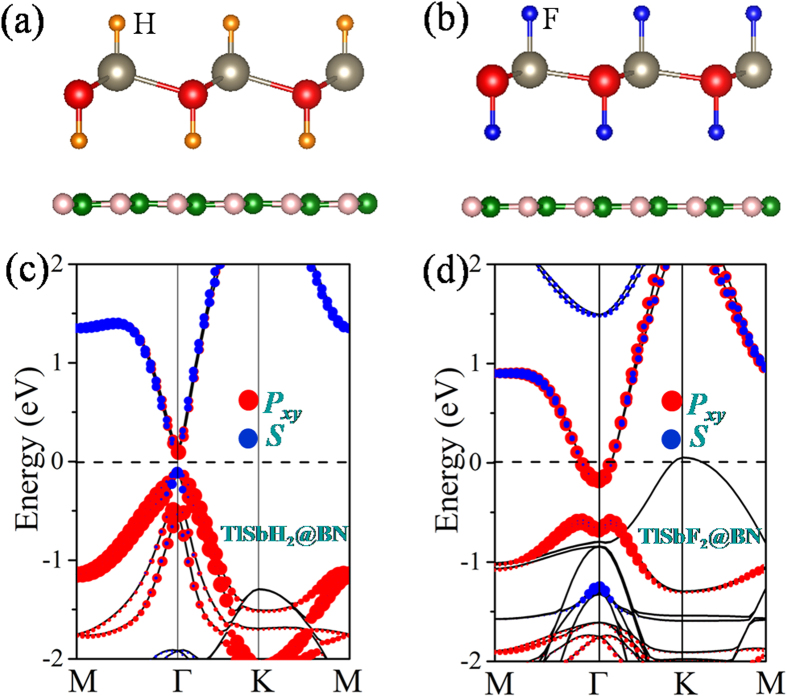
Crystal structures of TlSbX_2_ grown on BN substrate from the top and side view for (**a**) TlSbH_2_ and (**b**) TlSbF_2_. (**c**) TlSbH_2_ and (**d**) TlSbF_2_ correspond to the orbital-resolved band structures with SOC.

The substrate materials are inevitable in device application, thus a free-standing film must eventually be deposited or grown on a substrate. As a 2D large-gap insulator with a high dielectric constant, the BN sheet has been successfully used as the substrate to grow graphene or assemble 2D stacked nanodevices^40^,^41^. Thus, we use it as a substrate to support TlSbX_2_ films. Figure 6(a,b) show the geometrical structures of TlSbH_2_ and TlSbF_2_ on (2 × 2) BN sheet, where the lattice mismatch is only about 1.68% and 2.80%, respectively. After full relaxation with van der Waals (vdW) forces^42^, they almost retain the original structure with a distance between the adjacent layers of 3.35 Å. The calculated binding energy is about −69, −87, −98, −108, and −114 meV for TlSbH_2_, TlSbF_2_, TlSbCl_2_, TlSbBr_2_, and TlSbI_2 _per unit cell, respectively, showing that they are typical van der Waals heterostructures. The calculated band structure with SOC is shown in Fig. 6(c,d). In these weakly coupled systems, TlSbH_2_ on the BN sheet remains semiconducting, there is essentially no charge transfer between the adjacent layers, and the states around the Fermi level are dominantly contributed by TlSbH_2_. If we compare the bands of TlSbH_2_ with and without BN substrate, little difference is observed. Similar results are also found for all halogenated TlSbX_2_ films on BN substrate (see also Fig. S4), except that TlSbF_2_ on the BN sheet exhibits a metallic state. Evidently, all TlSbX_2_ films on BN substrate are robust QSH insulators.

## Conclusions

To conclude, on the basis of first-principles calculations, we predict a class of new QSH insulator of TlSbX_2_ (X = H, F, Cl, Br, I) films, with a sizable bulk gap (0.22 ~ 0.40 eV), allowing for viable applications in spintronic devices. Two mechanisms, type-I: SOC-induced *p-s-p* type TI (TlSbH_2_), and type-II: the chemical bonding induced *p-p-s* type TI (Halogenated ones) are obtained, significantly different from one in TISb monolayer. The topological characteristic of TlSbX_2_ films are confirmed by the Z_2_ topological order and topologically protected edge states. Furthermore, the band gap and topological phase transition could be tuned by the external strain and vertical *E*-field. When TlSbX_2_ deposited on BN substrate, both the band gaps and low-energy electronic structures are only slightly affected by the interlayer coupling from the substrate. These predicted QSH insulators and their vdW heterostructures may provide a platform for realizing low-dissipation quantum electronics and spintronics devices.

### Computational method and details

To study the structural and electronic properties of TlSbX_2_ (X = H, F, Cl, Br, I) films, our calculations were performed using the plane-wave basis Vienna *ab initio* simulation package known as VASP code^43^,^44^. We used the generalized gradient approximation (GGA) for the exchange and correlation potential, as proposed by Perdew-Burk-Ernzerhof (PBE)^45^, the projector augmented wave potential (PAW)^46^ to treat the ion-electron interactions. The energy cutoff of the plane waves was set to 500 eV with the energy precision of 10^−6 ^eV. The Brillouin zone was sampled by using a 21 × 21 × 1 Gamma-centered Monkhorst-Pack grid. The vacuum space was set to 20 Å to minimize artificial interactions between neighboring slabs. SOC was included by a second vibrational procedure on a fully self-consistent basis. The phonon spectra were calculated using a supercell approach within the PHONON code^47^.

## Additional Information

**How to cite this article**: Zhang, R. *et al*. Functionalized Thallium Antimony Films as Excellent Candidates for Large-Gap Quantum Spin Hall Insulator. *Sci. Rep.* **6**, 21351; doi: 10.1038/srep21351 (2016).

## Supplementary Material

Supplementary Information

## Figures and Tables

**Table 1:** Calculated structural parameters of the TlSbX_2_ (X = H, F, Cl, Br, I) films, including the lattice parameter *a*(Å), buckled height *h*(Å), bulk gap *E*_*g*_ (eV) and *E*_*Γ*_ (eV), while the *d*_TlSb_, *d*_Tl-X_, and *d*_Sb-X_ are the bond lengths of Tl-Sb, Tl-X, and Sb-X atoms, respectively (in Å).

Structure	*a*(Å)	*h*(Å)	*E*_*g*_(eV)	*E*_*Γ*_(eV)	*d*_*TlSb*_(Å)	*d*_*Tl-X*_(Å)	*d*_*Sb-X*_(Å)
*TlSb*	4.810	0.78	0.28	0.29	2.88	—	—
*TlSbH*_*2*_	4.911	0.87	0.22	0.25	2.97	2.13	1.78
*TlSbF*_*2*_	5.270	0.42	0.40	0.47	3.07	2.15	1.95
*TlSbCl*_*2*_	5.268	0.59	0.34	0.42	3.06	2.51	2.37
*TlSbBr*_*2*_	5.098	0.70	0.32	0.44	3.03	2.63	2.53
*TlSbI*_*2*_	5.050	0.78	0.29	0.49	3.02	2.82	2.74
