# Supplementary material for: Functionalized Thallium Antimony Films as Excellent Candidates for Large-Gap Quantum Spin Hall Insulator
Source: Sci Rep. 2016 Feb 17;6:21351. doi: 10.1038/srep21351 (PMC4756673; doi:10.1038/srep21351)
Supplement: Supplementary Information [file srep21351-s1.doc]

**Supplementary Information for:**

**Functionalized Thallium Antimony Films as Excellent Candidates for Large-Gap Quantum Spin Hall Insulator**

Run-wu Zhang,^a^ Chang-wen Zhang*,^a^ Wei-xiao Ji,^a^ Sheng-shi Li,^b^ Shi-shen Yan,^b^ Ping Li,^a^ and Pei-ji Wang,^a^

^a^ School of Physics and Technology, University of Jinan, Jinan, Shandong, 250022, People’s Republic of China

^b^ School of Physics, State Key laboratory of Crystal Materials, Shandong University, Jinan, Shandong, 250100, People’s Republic of China

* Correspondence and requests for materials should be addressed to: zhchwsd@163.com


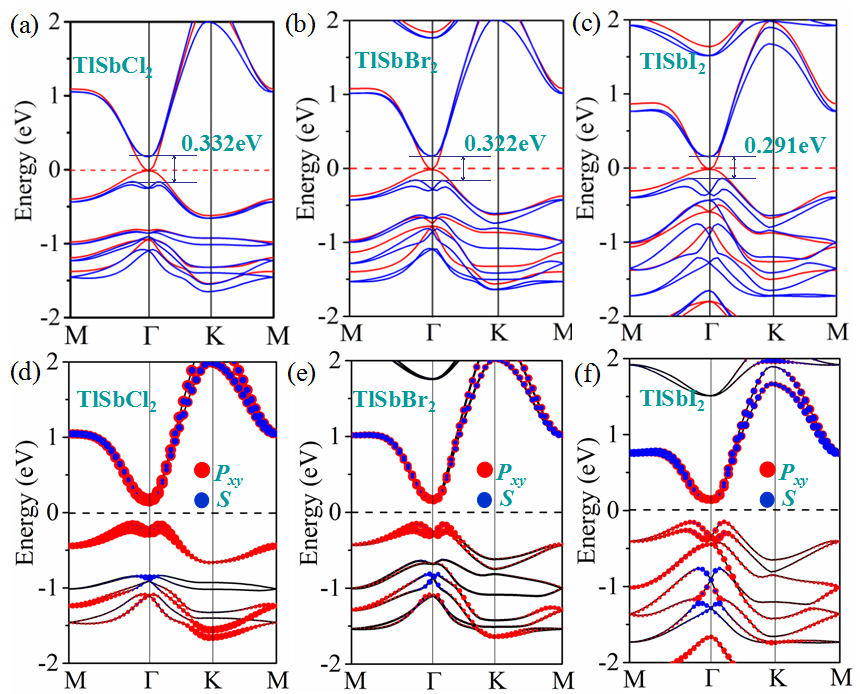


**Fig. S1** The calculated band structures of (a) TlSbCl_2_, (b) TlSbBr_2_ and (c) TlSbI_2_. The red lines correspond to band structures without SOC, and the blue lines correspond to band structures with SOC. Orbital-resolved band structures with SOC of (d) TlSbCl_2_, (e) TlSbBr_2_ and (f) TlSbI_2_, respectively. The blue dots represent the contributions from the *s* atomic orbital of Tl and Sb atoms and the red dots represent contributions from the *p*_x,y_ atomic orbitals of Tl and Sb atoms.

**
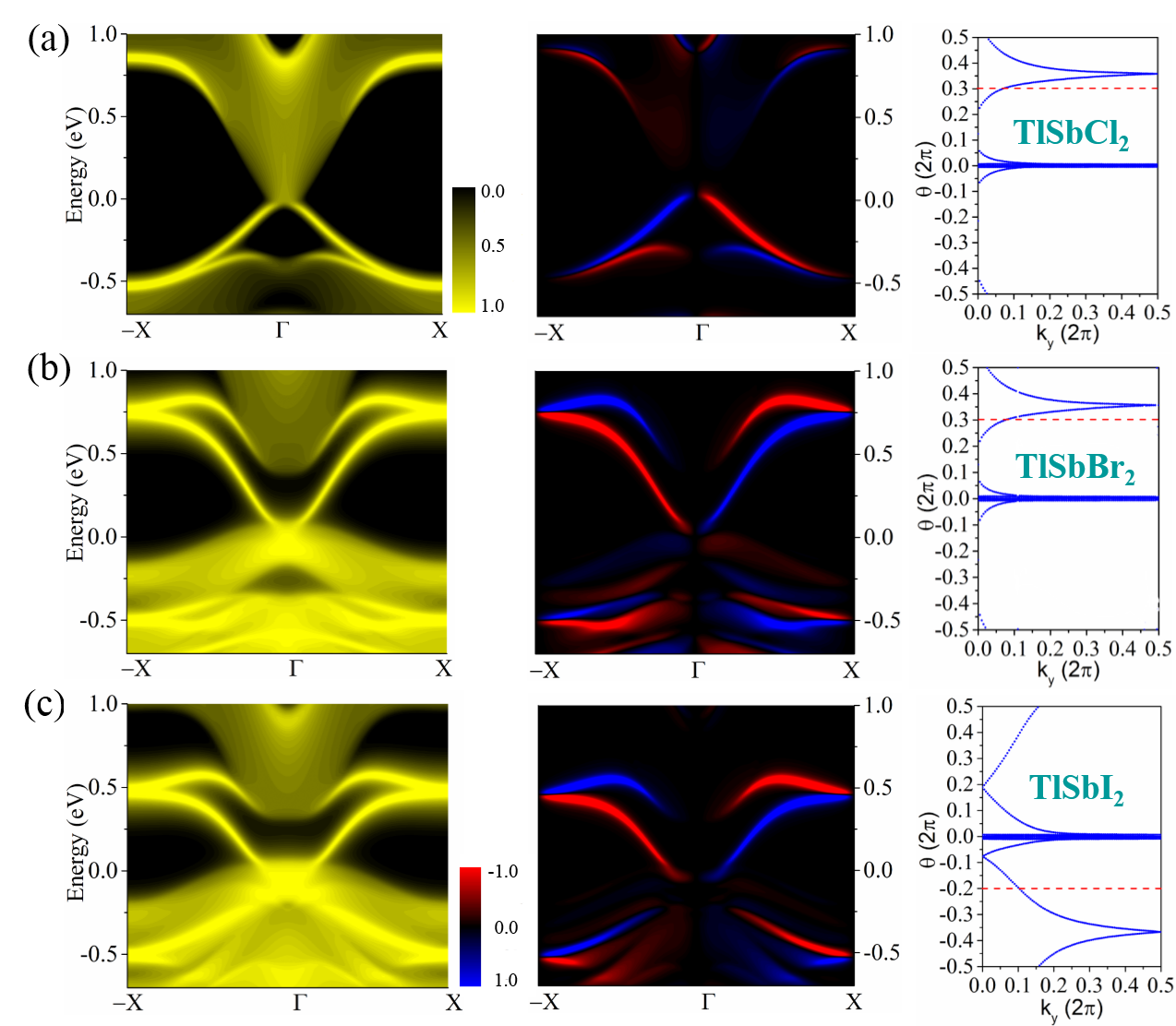
**

**Fig. S2** Total (left panel) and spin (right panel) edge density of states for (a) TlSbCl_2_, (d) TlSbBr_2_ and (e) TlSbI_2_. In the spin edge plot, red/blue lines denote the spin up/down polarization. Evolutions of Wannier centers along *k_y_* for (a) TlSbCl_2_, (b) TlSbBr_2_ and (c) TlSbI_2_. The evolution lines (blue dot lines) cross the arbitrary reference line (red dash line) parallel to *k_y_* an odd number of times, yielding Z_2_ = 1.

**
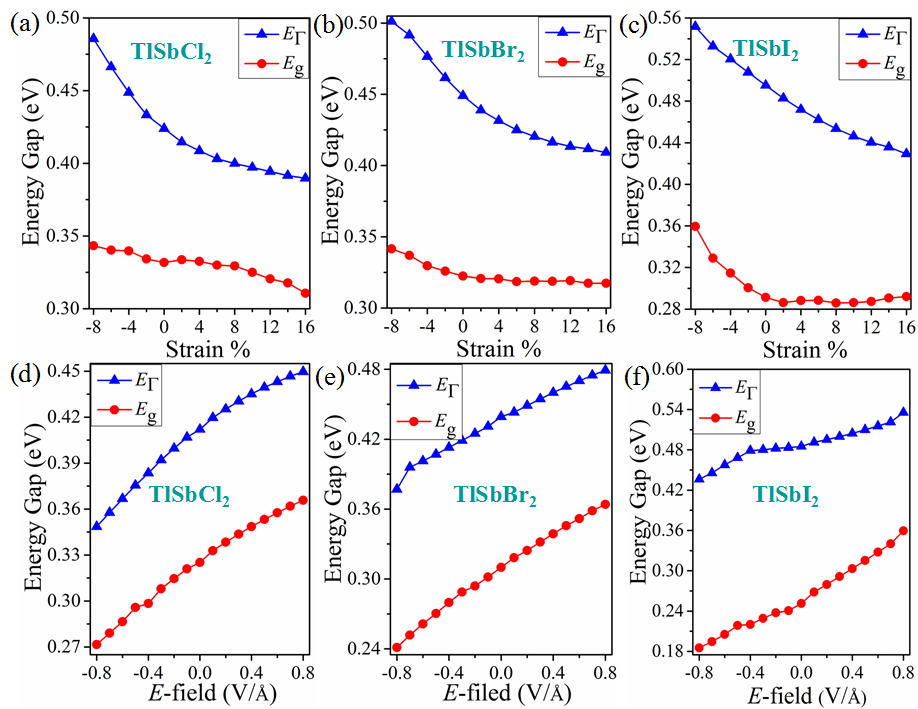
**

**Fig. S3** Strain and electric field dependencies of the global bulk-gap with SOC for (a) and (d) TlSbCl_2_, (b) and (e) TlSbBr_2_, and (c) and (f) TlSbI_2_, respectively.

**
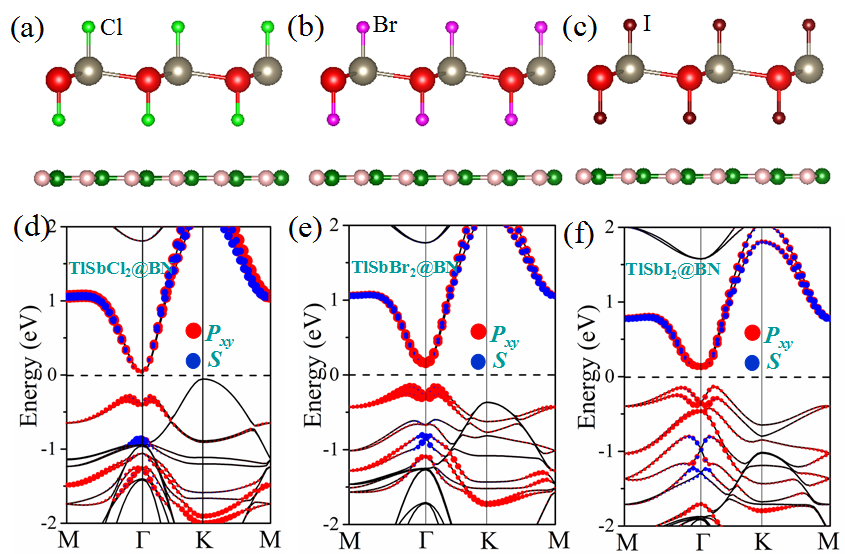
**

**Fig. S4** Crystal structures of TlSbX_2_ grown on BN sheet from the top and side view for (a) TlSbCl_2_, (b) TlSbBr_2_ and (c) TlSbI_2_. Orbital-resolved band structures with SOC are displayed in (d) TlSbCl_2_, (d) TlSbBr_2_ and (f) TlSbI_2_, respectively.
